# Next-Generation Anti-Angiogenic Therapies as a Future Prospect for Glioma Immunotherapy; From Bench to Bedside

**DOI:** 10.3389/fimmu.2022.859633

**Published:** 2022-06-10

**Authors:** Parisa Shamshiripour, Fahimeh Hajiahmadi, Shahla Lotfi, Niloofar Robab Esmaeili, Amir Zare, Mahzad Akbarpour, Davoud Ahmadvand

**Affiliations:** ^1^ Faculty of Medicine, Iran University of Medical Sciences, Tehran, Iran; ^2^ Department of Molecular Imaging, Faculty of Advanced Technologies in Medicine, Iran University of Medical Sciences, Tehran, Iran; ^3^ Department of Biomedical Engineering, Amirkabir University of Technology (Tehran Polytechnic), Tehran, Iran; ^4^ Department of Surgery, School of Medicine, Iran University of Medical Sciences, Tehran, Iran; ^5^ Advanced Cellular Therapeutics Facility, David and Etta Jonas Center for Cellular Therapy, Hematopoietic Cellular Therapy Program, The University of Chicago Medical Center, Chicago, IL, United States; ^6^ Immunology Board for Transplantation and Cell-Based Therapeutics (Immuno-TACT), Universal Science and Education Research Network (USERN), Tehran, Iran; ^7^ Neuroscience Research Center, Iran University of Medical Sciences, Tehran, Iran

**Keywords:** glioma, immunotherapy, anti-angiogenesis therapy, tyrosine kinase inhibitors, monoclonal antibodies, siRNAs

## Abstract

Glioblastoma (grade IV glioma) is the most aggressive histopathological subtype of glial tumors with inordinate microvascular proliferation as one of its key pathological features. Extensive angiogenesis in the tumor microenvironment supplies oxygen and nutrients to tumoral cells; retains their survival under hypoxic conditions; and induces an immunosuppressive microenvironment. Anti-angiogenesis therapy for high-grade gliomas has long been studied as an adjuvant immunotherapy strategy to overcome tumor growth. In the current review, we discussed the underlying molecular mechanisms contributing to glioblastoma aberrant angiogenesis. Further, we discussed clinical applications of monoclonal antibodies, tyrosine kinase inhibitors, and aptamers as three major subgroups of anti-angiogenic immunotherapeutics and their limitations. Moreover, we reviewed clinical and preclinical applications of small interfering RNAs (siRNAs) as the next-generation anti-angiogenic therapeutics and summarized their potential advantages and limitations. siRNAs may serve as next-generation anti-angiogenic therapeutics for glioma. Additionally, application of nanoparticles as a delivery vehicle could increase their selectivity and lower their off-target effects.

## Introduction

Brain gliomas are a major neurooncological challenge because of high mortality, morbidity and recurrence rates. Among the glial neoplasms of the brain, glioma grade IV or glioblastoma multiform (GBM) is the most frequent and deadliest. Despite the current advances in development of novel therapeutic strategies for gliomas, the prognosis of patients suffering high-grade gliomas is very poor. Up to present, aggressive surgery (ideally gross total resection of the tumor bulk), Temozolomide (TMZ) chemotherapy, and radiation therapy are the standalone and gold standard of care for GBM, based on the National Comprehensive Cancer Network (NCCN) guidelines. Meanwhile, many patients receiving the standard of care experience recurrence and disease-specific survival and progression-free survival for GBM patients are very poor. Extensively aggregating focally anastomosing capillaries forming glomeruloid vessel-like structures which are supported by basal lamina and pericytes and are devoid of astrocytic end-feet is a key histopathological characteristic of GBM (1). Hence, anti-angiogenic therapy is one of the well-known adjuvant therapy strategies for GBM. Proposing that vascular-reach tumor such as GBM depend on neovessel formation for survival and nutrient supply, inhibiting tumor angiogenesis is one of the key treatment strategies that could help combat glioma growth and also increase the patients’ quality of life due to symptom alleviation and reduction of peritumoral edema. Additionally, tumor aberrant angiogenesis supports the immunosuppressive tumor microenvironment (TME) in GBM and hence, reducing the angiogenic signals in the TME could enhance anti-tumor immune responses. However, the hypoxia caused by the severe reduction of tumor vasculature after anti-angiogenic therapy, contributes to activation of compensatory signals which resist against anti-angiogenic therapies and maintain tumor angiogenesis. Herein, we critically discuss the major contributing mechanisms to tumor angiogenesis and resistance to anti-angiogenic therapeutics and also discuss the novel advances in the field of designing anti-angiogenic therapeutics for GBM. We provide a detailed discussion on antibody-based anti-angiogenic therapies; small peptides; tyrosine kinase inhibitors and oligonucleotide-based therapeutics (e.g. aptamers and siRNAs) and critically review their potential challenges, safety, efficacy and future perspective. Furthermore, we explore the challenges of BBB for targeted brain delivery and strategies to overcome comprising passive and active targeting of both biological and synthetic nano-carriers.

## Biology of GBM Angiogenesis and Resistance Mechanisms to Anti-Angiogenic Therapy

**Figure 1:**
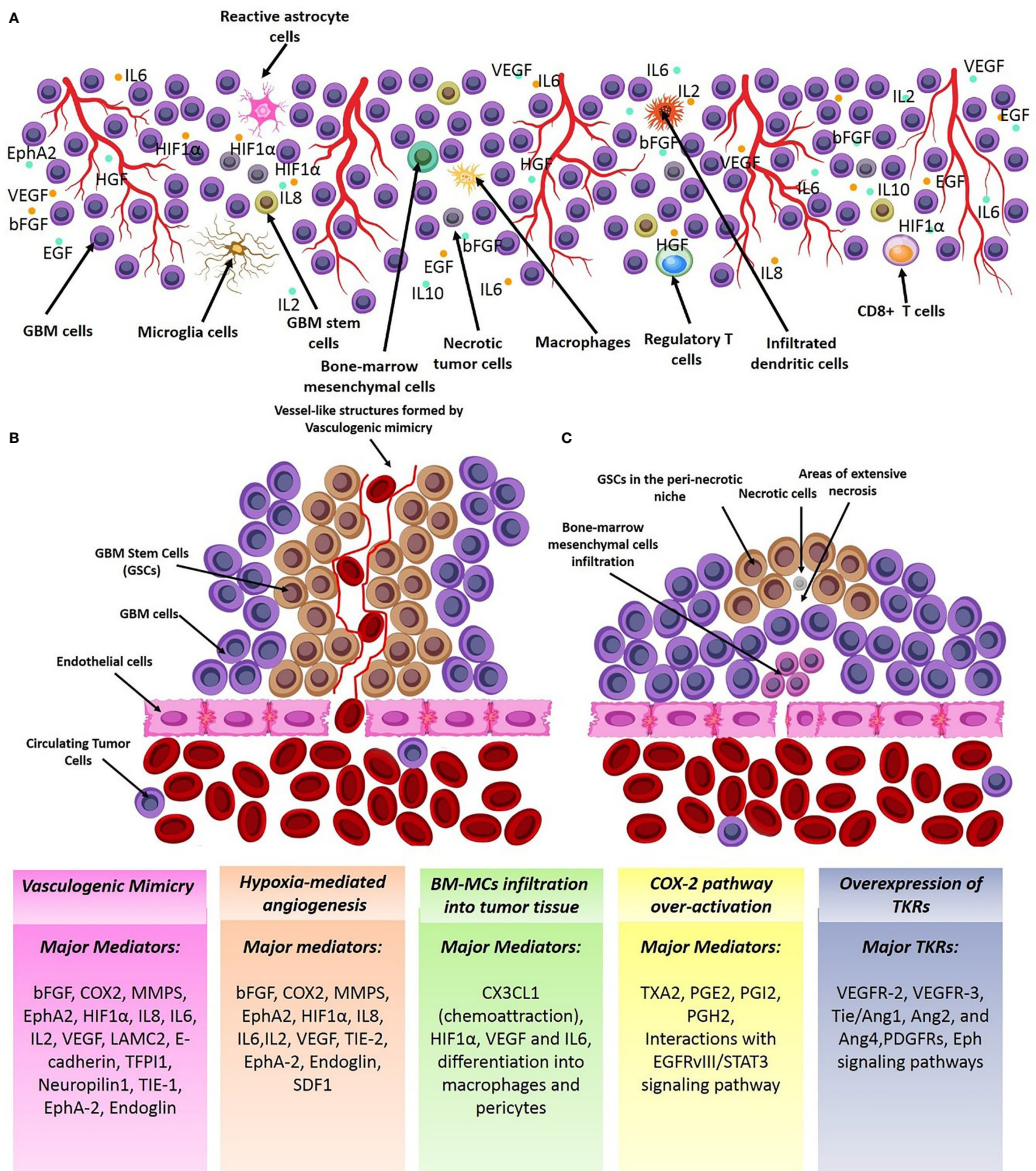
Major mechanisms of GBM angiogenesis. **(A)** Cellular schema of the angiogenic tumor microenvironment (TME) of GBM. GBM TME consists numerous cell sources (e.g. immune cells, GBM cells, astrocytes, macroglia/macrophages, and astrocytes) which support the angiogenic TME by overproducing angiogenic cytokines. **(B)** Vasculogenic mimicry; GSCs form vessel-like structures which invade to tumoral endothelia to get nutrient and oxygen supply. **(C)** Chemical attraction and infiltration of angiogenic clones of BMSCs in the tumor tissue by TME chemotactic signals. Some of the vectors used to design this figure were downloaded from Vecteezy under a free license.

**Figure 2:**
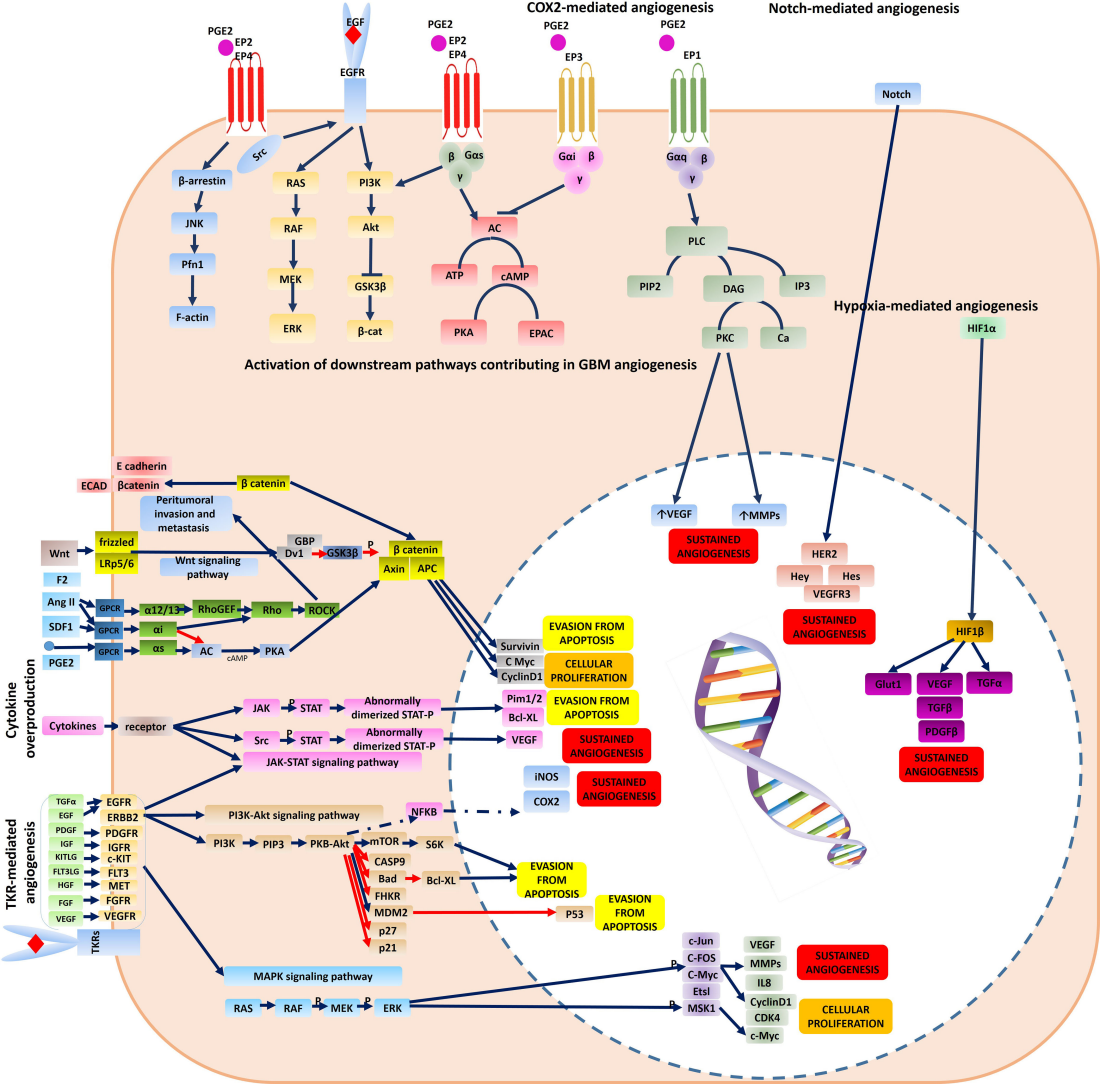
Molecular mechanisms contributing to tumor angiogenesis and resistance to anti-angiogenic therapy in a GBM cell. The major molecular pathways responsible for GBM angiogenesis comprise: (1) TKR-mediated angiogenesis *via* activation of downstream signaling pathways (PI3K-Akt signaling pathway, and MAPK signaling pathway) (2) cytokine-mediated angiogenesis *via* activation of downstream signaling pathways (JAK-STAT signaling pathway) (3) SDF1, ANG2 as compensatory mechanisms (4) Hypoxia-mediated angiogenesis *via* activation of HIF1α signaling pathway (5) COX2-mediated angiogenesis *via* activation of G-protein- coupled receptors mediating activation of protein-kinase C (PKC) and also its interactions with EGFR signaling (6) Notch-mediated angiogenesis. Some of the vectors used to design this figure were downloaded from Vecteezy under a free license.

Of all brain tumors, gliomas make up about 70% of brain neoplastic lesions. The prognosis of patients with high-grade gliomas is very poor despite development of advanced neurosurgical approaches. Glioma angiogenesis has long been considered to be a key controller of tumor progression and acquisition of aggressive phenotypes. Tumor aberrant angiogenesis was first described by Folkman et al. (2). Currently, several contributing cellular and molecular mechanisms have been proposed for incremental angiogenesis in GBM tumors, the most stated of which are as follows: (1) Hypoxia: The extensive cellular proliferation in tumoral bulk of the GBM, causes severe hypoxia, nutrient-deprivation and also induces secretion of angiogenic cytokines (**Figure 1**) and Matrix Metalloproteinases (MMPs). Consequently, neo-angiogenesis forming haphazard blood vessels lacking normal vessels structure speeds-up due to extracellular matrix (ECM) degradation. Quiescent endothelial cells (ECs) get activated *via* complex downstream signaling pathways induced by the TME cytokines, and ECs extensively proliferate and sprout in a complex TME comprising pericytes, reactive astrocytes, glioma-associated macrophages (GAMs), tumoral cells and ECs. Propagation of newly-formed vessel buds are enhanced by the interactions of binding proteins (e.g. αvβ5 and αvβ3 integrins) and furthermore, pericytes and smooth muscle cells eventually surround the newly lumenized tubes and stabilize their basement membranes. In the proangiogenic and proinflammatory TME of the GBM, extensive proliferation of ECs leads to formation of leaky and abnormal blood vessels. However, the haphazard vessels formed are not able to efficiently deliver the nutrients and oxygen demand of the proliferating tumor bulk and also are not efficient for delivering the chemotherapeutics. (2) Vasculogenic Mimicry (VM) of Cancer Stem Cells (CSCs): firstly, Maniotis et al. reported melanoma cells forming tube-like structure with no vascular endothelial cells containing red blood cells and this type of vessel formation was therefore named vasculogenic mimicry (3). Tumor-initiating cells have high dedifferentiation plasticity and can Trans-differentiate to vessel-like structures (identified by accumulation of RBCs and CD31/CD34-negative and PAS-positive cells) supported by glycoproteins comprising type I, IV, and VI collagen, and laminin Ln5 and its cleavage products, γ2x and γ2´ (4, 5). These vessel-like structures eventually merge with micro-vessels formed by angiogenesis or vascularization to retain blood supply and nutrient delivery and also play pivotal roles in tumor metastasis by shedding tumoral cells directly into the bloodstream. Epithelial-Mesenchymal Transition (EMT) and VE-cadherin/EphA2/MMP signaling pathway are key players to facilitate VM. Additionally, adenosine/STAT3/IL-6 pathway, MAPK/ERK pathway, Wnt/β-catenin, Notch, Wnt, Hedgehog, Hippo signaling pathway are also key triggers for VM due to their pivotal roles in generating CSCs. Hypoxic TME is a key trigger for GBM angiogenesis. The major contributing TME cytokines in VM are summarized in **Figure 1** (6). Over production of HIF1α in the TME increases BCL9 expression, mediating activation of β-catenin-mediated transcriptional activity at hypoxic tumor tissues, and facilitates VM. Tight junction proteins are negatively regulated by overexpression of CSCs markers (Twist and Snail) enhancing the migration capacity of endothelial cells (7). Moreover, the ECM is degraded by overproduction of matrix metalloproteinase such as MMP9 mediating EphA2/MMP signaling pathway. Afterwards, the newly-proliferated cancer stem cells form vessel-like structures in order to support tumor oxygen delivery. (3) Infiltration of Bone Marrow-Derived Mesenchymal Cells (BM-MCs): BM-MCs infiltrate into tumor tissues *via* chemo-attraction (e.g. by CX3CL1) and secrete pro-angiogenic and pro-inflammatory cytokines (e.g. HIF1α, VEGF and IL6). The recruited tumor-associated BMDCs may differentiate into macrophages and pericytes. Macrophages in the TME modulate the pro- and anti-angiogenic balance by cytokine production and pericytes, derived from PDGFRβ+ BMDCs, can enhance the ECs survival and also provide an extensive mechanical support to maintain the vessels (**Figure 1**) (8). (4) Over-activation of Cyclooxygenase-2 (COX-2) hydroperoxidase pathway: COXs, checkpoint enzymes of prostanoids production, have two mammalian isoforms. COX-1 regulates the homeostatic synthesis of prostanoids expressed in the most tissues and retains the physiological functions of prostanoids at target organs. COX-2, also known as prostaglandin G/H synthase, is expressed at extremely low levels in physiological circumstances, and the robust increase in COX2 expression reflects severe inflammatory responses to tissue injuries and other detrimental stimuli such as tumorigenesis. COX2 activation results in an eventual overproduction of prostaglandin E2 (PGE2), thromboxane A 2 (TXA2) and prostaglandin I2 (PGI2). TXA2 facilitates ECs migration and proliferation and PGI2 is involved in multiple angiogenesis-related processes (e.g. ECs sprouting, ECs proliferation and vessel permeability; **Figure 2**). PGE2 facilitates glioma angiogenesis *via* protein kinase C activation (PKC) by activating G-protein-coupled receptors. Additionally, the interactions of Epidermal Growth Factor Receptors (EGFR)/Signal transducer and activator of transcription 3 (STAT3) or the epidermal growth factor receptor variant III (EGFRvIII)/STAT3 signaling axes with COX2 downstream pathways contributes to glioma angiogenesis (**Figure 2**) (9). (5) Overexpression of tyrosine kinase receptors (TKRs): Over-activation of TKRs are thought to be of the key players in oncogenesis. Major TKRs families which are extensively involved in tumor angiogenesis are thought to be the VEGF receptors (VEGFRs), the Tie receptors platelet-derived growth factor (PDGF) receptors and Eph receptors. VEGFR-2 and VEGFR-3 act in order to facilitate and drive angiogenesis, whereas VEGFR-1 restricts the angiogenic response and is said to be a key player in tissue remodeling acting to recruit macrophages. Under physiological circumstances, stimulation of VEGFR-2 contributes to angiogenesis of blood vascular ECs, however, activating VEGFR-3 elicits a similar response for lymphatic ECs. During the cancer pathogenesis, VEGFR2 is extremely overexpressed. Tie2/Ang1, Ang2, and Ang4 interactions also play pivotal roles in EC survival, stabilization and remodeling of blood and lymphatic vessels. The PDGF receptors mediate vascular wall stabilization by mural cells (e.g. pericytes and smooth muscle cells), and the Eph receptors contribute in determining arterial versus venous identity. The TKRs downstream signaling pathways contributing to glioma aberrant angiogenesis are depicted in detail in **Figure 2**. Over-activation of mitogen-activated protein kinase (MAPK) and Phosphoinositide 3-kinase (PI3K)-Akt signaling pathways eventually result in sustained angiogenesis, cellular proliferation and evasion from apoptosis (10).

Although the normalized tumor vasculature after anti-angiogenic therapy with a high dose interrupted protocol which is conventionally used as an adjuvant therapy for GBM with Tyrosine Kinase Inhibitors (TKIs) or monoclonal antibodies (mAbs) provides a transient window period for more efficient chemotherapy, high dose systemic consumption of mAbs or TKIs can cause tumor escape and acquisition of resistance to anti-angiogenic therapies (**Figures 1**, **2**). Previous studies have identified several mechanisms for tumor escape from anti-angiogenic therapy which include: (1) over-activation of compensatory angiogenic signaling pathways: In the hypoxic GBM TME with few remaining vessels after angiogenesis blockade, cells rewire their signaling pathways to activate compensatory signals comprising the Hypoxia-inducible factor signaling pathway, Notch signaling pathway, and Ang2/Tie2 signaling pathway (**Figure 2**). (2) Immunological escape: Briefly, pro-angiogenic signals produced by TIE2+ monocytes, GAMs, reactive astrocytes and CD11b+ myeloid cells, neutrophils and T helper-17 cytokines enhance infiltration of the pro-angiogenic clones of BM-DSCs and result in tumor angiogenesis. (3) Increased pericyte coverage: after anti-angiogenic therapy, extensive pericyte coverage may also be another contributing mechanism to maintain the survival of ECs. (4) Vessel co-option and perivascular invasion: Invasion of tumoral cells to co-opt with the vessels in the tumoral tissues is a well-known characteristic of aggressive tumors which facilitates nutrient delivery and oxygenation of the rapidly-expanding tumor tissue. The exact molecular mechanisms mediating vessel co-option are not yet fully-described however previous evidence highlights the role of Bradykinin/bradykinin receptor-2 (B2R) signaling pathway, CXCR4/SDF-1α pathway, MDGI/FABP3 signaling pathway, EGFRvIII signaling pathway, and Olig2/Wnt7a signaling pathway (11). It is noteworthy to note that increased pericyte coverage in the co-opted blood vessels supports survival of ECs under anti-angiogenic therapy by promoting an autocrine VEGF-A signaling and consequently, vessel co-option was previously noted as an indicator of poor clinical response to anti-angiogenic therapy in many cancers including breast, colorectal, lung and pancreatic cancer, GBM, melanoma, hepatocellular carcinoma, and renal cell carcinoma (12). Hence, the modulating molecular targets of vessel-cooption serve a potent future perspective for generating more efficient anti-angiogenic drugs for cancer therapy and also as a mechanism to enhance tumor chemo-sensitization. Co-option of tumoral cells to tumor vessels is also another compensatory mechanism to get oxygen and nutrient supply which supports the survival of tumoral ECs. (5) VM; as previously described. (6) Autophagy process: Both selective and non-selective autophagy mechanisms are ways to provide energy for tumoral cells in order to maintain their survival under hypoxic or anoxic conditions through both HIF-1 dependent/independent mechanisms. A well-known strategy to overcome anti-angiogenesis therapy resistance is to pursue low dose and continuous inhibition rather than disrupted high dose consumption. A future perspective therefore proposed is to use slow-releasing nanoparticles as vehicles for anti-angiogenic compounds.

## Optimization Strategies for Anti-Angiogenesis Drug Designing

The primary objective of anti-angiogenic therapy for GBM is to normalize the tumor vasculature rather than eliminating all the tumoral vessels. Normalized tumor vasculature serves as a window for more effective chemotherapy and enhance tumor delivery of therapeutic agents. An ideal anti-angiogenic drug should have the following characteristics: (1) Target multiple signaling pathways (2) cause minimal drug-induced resistance (3) increase endogenous anti-angiogenesis substances (4) have minimal off-target effects (5) high selectivity (6) and limited systemic toxicity. Anti-angiogenic therapeutics are categorized as tabulated in **Table 1**.

**Table 1:** Major categories of anti-angiogenic immunotherapeutics.

Anti-angiogenic immunotherapeutic	Major category	Examples
Intracellular Tyrosine Kinase Inhibitors (TKIs)	TKIs	mTOR inhibitors, protein kinase C inhibitors
Membrane TKIs		Sunitinib, Sorafenib
Ligand TKIs		VEGF inhibitors such as Bevacizumab; also categorized as a mAB
decoy receptors	decoy receptors	aflibercept
Matrix metalloproteinase inhibitors (MMPIs)	MMPIs	Marimastat
matrix-derived inhibitors	Endogenous angiogenic substance inhibitors	Konstatin,thrombospondin1-2, endostatin, endorphin, arsenic
non-matrix-derived inhibitors		angiostatin, antithrombin, TIMP 4, vasostatin
integrin antagonists	integrin antagonists	Vitaxin (integrin α5β3 mAB), Anti-integrin α5β1 blocking peptides, Cilengitide (integrin α5β3 and integrin α5β5; a cyclic RGD pentapeptide),
Cytokine/chemokine inhibitors	Cytokine/chemokine inhibitors	tumor necrosis factor (TNF) inhibitors, IL2 inhibitors, or α/β interferon (INF α/β) inhibitors
aptamers	aptamers	Pegabtanib
Monoclonal antibodies (mAbs; targeting angiogenic cytokines or TKRs)	mAbs	Tanibirumab, Cetuximab, Onartuzumab

## Anti-Angiogenic mAbs; Current Status, Challenges and Future Perspective

**Figure 3:**
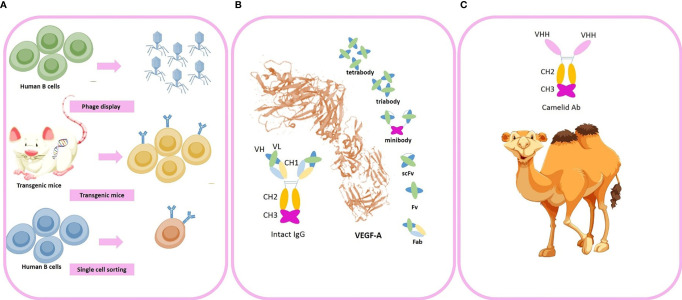
Novel advances in generation of anti-angiogenic monoclonal antibodies (mAbs). **(A)** Novel strategies used for industrial production of engineered fully-humanized mAbs comprising (1) phage display technologies (2) transgenic mice (3) single-cell sorting. **(B)** An intact mAb and antibody fragments (i.e. minibody, tirabody, tetrabody, and scFv) **(C)** camelid antibodies. Some of the vectors used to design this figure were downloaded from Vecteezy under a free license.

Currently, the clinical applicability of a large number of mAbs for GBM is under evaluation however major challenges exist to optimize the efficacy of anti-angiogenic therapy with mAbs which include: (1) Low tumor accumulation rates: low absolute tumor accumulation of the large molecules of intact mAbs are due to increased interstitial fluid pressure (IFP). The increased IFP and extensive peritumoral edema impair the trans-capillary transport of mAbs into the tumor tissue. High IFP slows the diffusion constants and forms a “binding site barrier” that causes uneven tumor penetration and hence mAbs tend to bind to the first antigen molecules they encounter. (2) Low concentration in tumor tissue due to the Blood-Brain Barrier (BBB): Prolonged high dose consumption of mAbs to overcome the BBB challenge insert systemic AEs and enhances acquisition of resistance to anti-angiogenic therapy due to extensive tumor hypoxia after angiogenesis blockade. (3) Long circulation time (days to weeks) resulting in dose-limiting toxicities (4) slow tumor uptake (5) heterogeneity in the expression of targeting antigens in the tumor tissue. Due to the aforementioned limitations of intact mAbs, there is a rising tendency to use antibody fragments with smaller molecular sizes (e.g. minibodies, diabodies, single-chain fragment variable, camelid antibodies, and small peptides) for future clinical applications. As a future perspective, camelid antibodies, also named as noanobodies or single-domain antibodies, are completely devoid of light chain and have only one single VH domain termed VHH in the antigen binding regions (13). Previously, we have successfully generated combined Mucin-1 (MUC1)-specific nanobody-tagged poly-ethylene glycol (PEG)-polyethylenimine polyplex targeting and transcriptional targeting of tBid transgene for directed killing of MUC1 over-expressing tumor cells (14). Production of camelid antibodies is a future perspective for antibody-based knockdown of tumor angiogenesis by phage display technologies with particular advantages comprising (1) small molecular size of about 10-15 KDs (2) robust kinetics and behavior (3) high affinity (4) high specificity and (5) better tissue penetration due to smaller size and (6) ability to deliver therapeutic cargos. Investigating the *in-vivo* efficacy of nano-bodies to successfully penetrate the BBB and deliver their cargo to the region of interest in the brain with high affinity for tumoral cells is a potential future perspective. Muruganandam et al. reported that two llama single-domain antibodies were selected, sequenced, subcloned, and expressed as fusion proteins with c-Myc-His5 tags which selectively bind to human cerebromicrovascular endothelial cells and transmigrate across an *in vitro* human BBB model (15). Additionally, Wouters et al. also reported successful generation of an anti-transferrin receptor nano-body that can reach the brain *via* receptor-mediated transcytosis after peripheral administration (16). Moreover, Li et al. also reported successful generation of two novel single-domain antibodies (VHHs or nano-bodies) against extracellular amyloid deposits and intracellular tau neurofibrillary tangles and reported gradual extravasation of the VHHs across the BBB, diffusion in the parenchyma and labeling of amyloid deposits and neurofibrillary tangles in transgenic Alzheimer’s disease mice models (17). Moreover, Farrington et al. (18), M Vandesquille et al. (19), and Rutgers et al. (20) also reported that camelid antibodies pass through the BBB. In a review by GAO Et Al the potential mechanisms by which the nano-bodies pass through the BBB are completely discussed (21). We are currently investigating the BBB penetration of some VHH clones by phage display strategy as an experimental project and aim to examine the efficacy of VHH nano-bodies as carriers for brain delivery as a future perspective. Despite previous evidence suggesting that nano-bodies may cross the BBB by direct penetration or RMT, yet future research may shed light to the exact molecular mechanisms mediating BBB penetration of VHHs and their efficacy to target tumoral tissues in the brain. Novel technologies developed for high-yield production of recombinant mAbs by cloning of immunoglobulin gene segments and producing libraries of antibodies (e.g. repertoire cloning, CRISPR/Cas9 and phage display) has attracted much attention in the recent years compared to the traditional methods (e.g. chimeric antibodies, and hybridoma technologies) Traditional methods are less efficient and may cause adverse events (AEs) such as human anti-mouse antibodies (HAMA) formation. Consequently, phage display technologies can be a potential future perspective to cross the blood-brain-barrier with nano-ligand drug carriers in clinics to optimize the drug delivery process for neurological disorders and brain tumors (22). The aforementioned technologies enables the scientific committee to engineer the mAbs with modified amino acid (AA) sequences to achieve the desired characteristics. Production of fully-humanized antibody fragments with modifiable AA sequences are the goal for novel anti-body-based products which can be achieved by newer antibody engineering techniques (e.g. phage display, transgenic mice and single B cell cloning; **Figure 3**). Small peptides are also potentially more advantageous to intact mAbs due to faster clearance rates and tumor penetration. However, one of the major drawbacks of peptides is that a slight change in their AA composition causes major conformation modification which results in huge changes in their relative affinity. Consequently, they are relatively less potent for designing novel therapeutic conjugates than mAbs.

## CLINICAL TRIAL PIPELINES OF ANTI-ANGIOGENIC mAbs/fusion proteins FOR GBM

As summarized in **Table 2**, the majority of previous evidence suggests poor clinical applicability of mAbs for GBM. However, Bevacizumab (BV), a humanized anti-VEGF mAB targeting circulating VEGF, is now broadly used as an FDA-approved adjuvant immunotherapy for recurrent GBM. A major concern for using BV in patients suffering GBM at childbearing ages is that it may impair fertility. Other serious systemic adverse events (AEs) of BV include gastrointestinal perforation, thromboembolic events, renal injury, and impairment of wound healing process which may increase the risk of post-surgical infections, Posterior Reversible Encephalopathy Syndrome (PRES), congestive heart failure and hypertension. Aflibercept: Aflibercept is an IV-injected soluble decoy receptor (a decoy fusion protein of domain 2 of VEGFR-1 and domain 3 of VEGFR-2 with the Fc fragment of IgG1) binds to VEGF-A, VEGF-B, and PGF with greater affinities than their native receptors (e.g.VEGFR family). Hence, it traps the soluble VEGF preventing its interaction with VEGFR family to activate downstream angiogenic pathways. The phase II trials of Aflibercept in patients with recurrent GBM reported moderate toxicity, including fatigue, hypertension, lymphopenia, CNS ischemia and systemic hemorrhage. In addition, in another trial of TMZ-resistant malignant gliomas, moderate toxicity was also reported with major adverse events being fatigue, hypertension, hand-foot syndrome, lymphopenia, thrombosis, proteinuria, CNS ischemia and systemic hemorrhage. Further, a phase I clinical evidence suggested that Aflibercept in combination with TMZ was well tolerated and the dose-limiting toxicities reported were thrombotic microangiopathy and thrombocytopenia in the dose escalation study. Although the monotherapy of Aflibercept may cause moderate systemic toxicities, potential advantages of adding Aflibercept to TMZ chemotherapy still needs further clinical investigations (23–26). Tanibirumab: In a phase I investigation, Tanibirumab was considered tolerable and had modest clinical efficacy for refractory solid tumors. Furthermore, the results of a phase IIa clinical study of Tanibirumab in patients with recurrent GBM revealed that Tanibirumab is safe and a common AE of Tanibirumab was cutaneous hemangiomas (27, 28). Cetuximab: Cetuximab is an EGFR inhibitor, a fully humanized mAB, with FDA-approval for patients with K-Ras wild-type, EGFR-expressing colorectal cancer, metastatic colorectal cancer and advanced squamous cell carcinoma of the head and neck in combination with radiation therapy. Results of a phase II clinical trial suggested that cetuximab had both minor toxicities and minor clinical benefits in progressive malignant gliomas (29). Two serious AEs of Cetuximab reported in patients with head and neck squamous were heart attacks and sudden deaths. Onartuzumab: Onartuzumab is a fully-humanized and monovalent antibody against c-met. A phase II trial reported adding Onartuzumab to BV had no further clinical benefits compared to BV alone (30).

**Table 2:** Clinical trials on mAbs/fusion proteins for GBM.

mAB	Co-therapy	Target	Phase	Antibody type	Ref.
Aflibercept	–	VEGF-A, VEGF-B, PGF	II	Fully-humanized IgG	(23, 24)
	+TMZ		I		(25)
	+ radiation therapy+ TMZ		I		(26)
Tanibirumab	–	VEGFR2	II	Fully-humanized IgG	(27)
			I		(28)
Cetuximab	–	EGFR	II	Fully-humanized IgG	(29)
Onartuzumab	+Bevacizumab	c-MET	II	Fully-humanized IgG	(30)

## Anti-Angiogenic Tyrosine Kinase Inhibitors (TKIs); Current Status, Challenges and Future Perspective

TKs are a group of phosphorylating enzymes which activate a variety of downstream pathways resulting in a biological response (e.g. cellular proliferation, differentiation, migration, survival, vessel formation or permeability). TKs can be further categorized as Receptor-Tyrosine Kinases (RTKs) and non-Receptor-Tyrosine Kinases (nRTKs). RTKs transduce extracellular signals into cells, while nRTKs reform intracellular communications. The downstream pathways of the overexpressed TKRs result in glioma angiogenesis and proliferation (**Figure 2**). Up to the present, a large number of TKIs are under clinical investigation for GBM. TKIs, as small hydrophobic molecules, can pass through the cellular membranes and inhibit the functions of multiple downstream pathway, whereas mAbs are extracellular antagonists of specific protein targets. Therefore, the majority of anti-angiogenic TKIs have multiple targets of several signaling pathways (e.g. VEGFRs EGFRs, FGFRs and PDGFRs) despite mAbs. Therefore, TKIs are more potent to reduce tumor angiogenesis than single-targeted blockade with mAbs. However, due to limited selectivity of TKIs, the off-target effects and systemic AEs are major challenges of TKIs. Despite the overproduction of TKRs in cancer cells and tumor ECs, TKs are expressed in lower levels in all cells. Consequently, inhibition of TKs can result in the impairment of important hemostatic or endocrine organs functions (e.g. thyroid gland or kidney). Nephrotic syndrome and hypothyroidism are rare but possible off-target effects of TKIs. Moreover, TKIs administration impairs the wound healing process due to a significant reduction in growth factors and may also cause bleedings due to impaired platelet interaction with ECs. In addition, the tumor heterogeneity affects the efficacy of TKIs. Taken together, accumulating evidence suggests that there are still many concerns about TKIs, including systemic AEs and low selectivity. Previously, it was taught that TKIs and mAbs do not insert cytotoxic effects on normal endothelia due to the quiescent state of adulthood ECs and only target tumor angiogenesis. However, some further investigations unveiled that anti-angiogenic therapy reduces survival and renewal capacity of normal ECs *via* growth factor signaling pathways. Hence, the development of novel anti-angiogenic agents with lower off-target effects can help reduce the side effects of systemic administration of TKIs or mAbs.

## Clinical Trial Pipelines of Anti-Angiogenic TKIs for GBM

**Table 3** summarizes some previous clinical trial pipelines of Antiangiogenic TKIs on GBM. Axitinib: Axitinib showed a manageable toxicity profile and the most frequent grade III/IV AEs were fatigue, diarrhea and oral hyperesthesia. Axitinib had objective response rates as a monotherapy compared with BV or lomustine (28% in axitinib-treated individuals and 23% in BV or lomustine-treated group) in a phase II trial. Another phase II trial suggested that Axitinib increases the response rate and progression-free survival in recurrent GBM, but the combination therapy of lomustine and Axitinib did not show any promising priorities compared to Axitinib monotherapy. The results of another phase II trial testing clinical efficacy and safety of Axitinib+Avelumab were not justifying for further clinical investigations (31–33). Cabozantinib: Phase I clinical investigations of Cabozantinib concurrent with chemradiation therapy in newly diagnosed patients with high‐grade gliomas were well-tolerated and also showed promising results (34). Lenvatinib: in a phase I/II study for recurrent and refractory pediatric CNS tumors, the clinical efficacy and safety of Lenvatinib+ everolimus was investigated (35). Nintedanib: phase II clinical study of Nintedanib in recurrent high-grade gliomas showed promising results regardless of previous BV therapy. However, in another phase II clinical trial, Nintedanib showed minimal clinical anti-tumor activity despite its perfect safety profile with no grade III/IV AEs (36, 37). phase I/II trial in adult patients with relapsed malignant glioma, and the results showed limited efficacy. Also, monotherapy of Pazopanib did not associate with any significant survival benefits in a phase II investigation in patients with recurrent GBM (38–40). Sunitinib: monotherapy of Sunitinib showed insufficient activity as a monotherapy regimen in recurrent high-grade gliomas. The combination therapy of Sunitinib and Irinotecan showed moderate toxicity and limited anti-tumor activity (41–47). Ponatinib: Ponatinib administration in patients with BV-refractory GBMs showed minimal clinical efficacy (48). Regorafenib: The results of comparing Regorafenib with Lomustine in patients with relapsed GBM was promising with an encouraging overall survival benefit (49). Sorafenib: Sorafenib combined with radiation therapy and TMZ showed significant AEs and resulted in moderate clinical outcomes (50, 51). Vandetanib: Seizures were a major concern as a serious AE in Vandetanib monotherapy, and Vandetanib did not show significant anti-tumor activity in patients with recurrent malignant glioma (52–54).

**Table 3:** Clinical trials on TKRs for GBM.

TKI	Co-therapy	Target	Phase	Ref.
Axitinib	–	VEGFR1, VEGFR2, VEGFR3	II	(31)
	+ lomustine			(32)
	+ avelumab			(33)
Cabozantinib	+TMZ+RT	RET, MET, VEGFR1, VEGFR2, VEGFR3, KIT, TRKB, FLT-3, AXL, TIE-2	I	(34)
Lenvatinib	+ everolimus	VEGFR1, VEGFR2, VEGFR3, FGFR1, FGFR2, FGFR3, PDGFRα, KIT, RET	I/II	(35)
Nintedanib	–	FGFR1, FGFR2, FGFR3, PDGFRα/β, VEGFR1, VEGFR2, VEGFR3, FLT3	II	(36, 37)
Pazopanib	+ lapatinib	VEGFR1, VEGFR2, VEGFR3, PDGFRα/β, FGFR 1/3, KIT, LCK, FMS, Itk	I/II	(38, 39)
	–		II	(40)
Sunitinib	–	PDGFRα/β, VEGFR1, VEGFR2, VEGFR3, c-KIT, FLT3, CSF-1R, RET	II	(41–45)
	+irinitecan		I	(46, 47)
Ponatinib	–	BCR-ABL, BCR-ABL T315I, VEGFR, PDGFR, FGFR, EPHR, SRC family kinases, KIT, RET, TIE2, FLT3	II	(48)
Regorafenib	–	VEGFR1, VEGFR2, VEGFR3, BCR-ABL, B-RAF, B-RAF(V600E),c-KIT, PDGFRα/β, RET, FGFR1/2, TIE2, Eph2A	II	(49)
Sorafenib	+RT*	B/C-RAF, B-RAF(V600E), KIT, FLT3, RET, VEGFR1, VEGFR2, VEGFR3, PDGFRβ	I/II	(50)
	–			(51)
Vandetanib		\ EGFR, VEGFR1, VEGFR2, VEGFR3, RET, BRK, TIE2, EPHRs, SRC kinases	I/II	(52)
	+RT*		I/II	(53)
	fractionated radiosurgery			(54)

*RT, Radiation therapy; VEGFR, VEGF receptor; PDGFR, Platelet-derived growth factor receptor; CSFR, Colony stimulating factor receptor.

## Anti-Angiogenic Aptamers

Aptamers are single-stranded short oligonucleotides with architectural folding to bind their targets (mostly proteins) with high specificity and affinity. Aptamers can be designed to target a wide range of biological targets (e.g. whole cells, nucleic acids, proteins, and peptides). Compared to mAbs, aptamers are advantageous in many aspects, which comprise (1) minimal immunogenicity (2) minimized toxicity (3) easy and fast *in-vitro* production without need for hosting animals (4) smaller size (8-15KD compared to 150 KD for mAbs) (5) higher tumor permeability (6) easy site-directed modifications (7) ability to be conjugated with broad ranges of tags (8) high chemical compatibility in organic and biological solutions (pH ranges:4-8.5 active temperature up to 95°C) and (9) lower cost. Design and discovery of aptamers is performed by Systematic Evolution of Ligands by Exponential Enrichment (SELEX) strategy, which includes a random synthesis step followed by selection, amplification and mutation steps. Potential challenges faced for clinical applications of aptamers are: (1) degradation by endogenous nucleases resulting in low stability in biofluids which can be further improved by backbone modifications (e.g. sugar modifications) or using spiegelmer^®^s. (2) Relatively high renal filtration rates. A possible strategy to overcome this challenge is PEGylation. The first anti-angiogenic aptamer FDA-approved for age-related macular degeneration was Pegaptanib-sodium (Macugen; Pfizer/Eyetech) (55). As a future perspective, aptamers can be used as theranostic agents delivering the cargo of interest to tumor site. Yet, further clinical investigations are required to shed light on the efficacy and safety of anti-angiogenic aptamers for GBM.

## The BBB Hurdle; Strategies to Overcome for GBM Anti-Angiogenic Therapy

**Figure 4:**
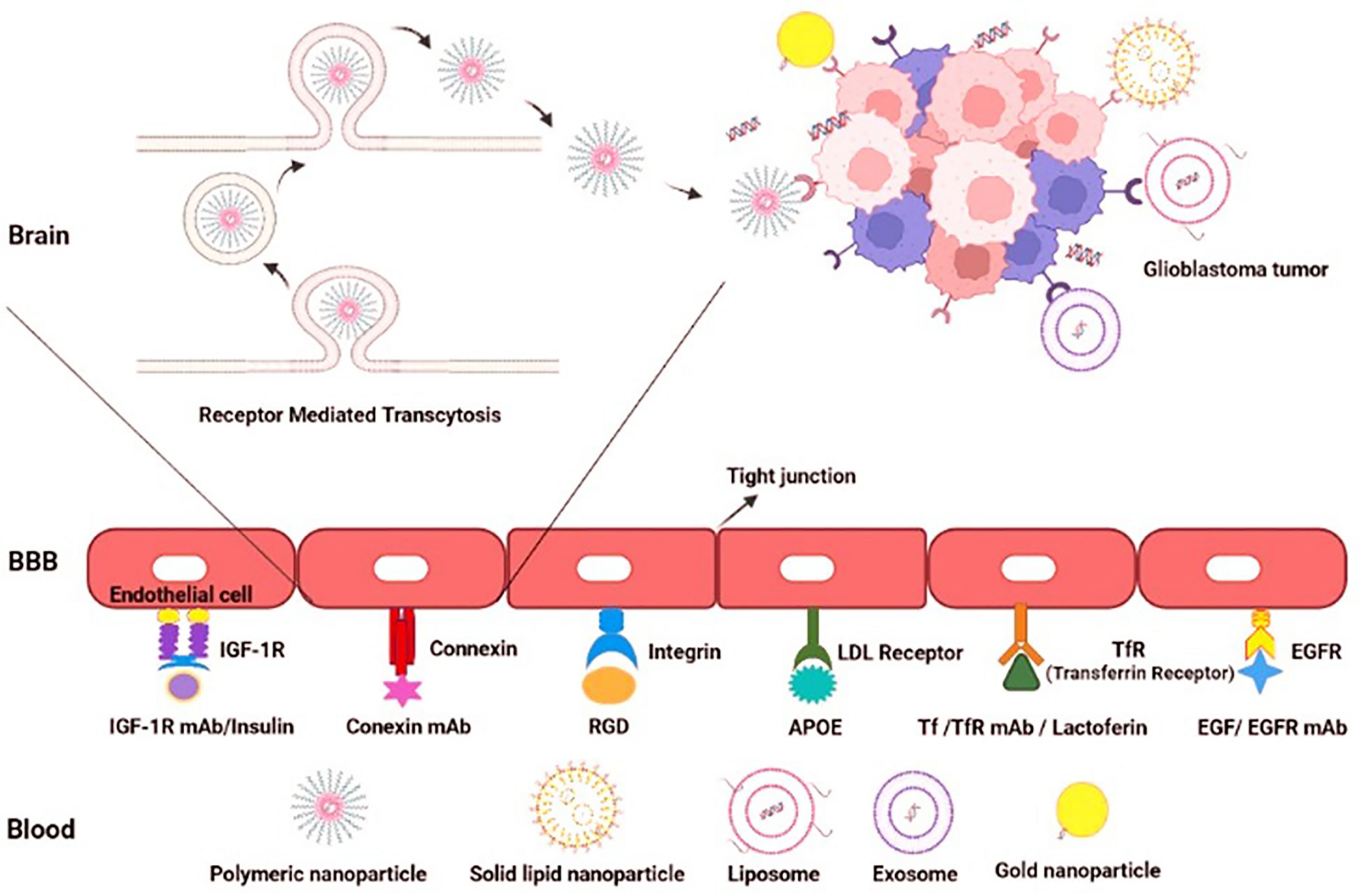
Active targeting to overcome the BBB hurdle for drug delivery in GBM. Active targeting using receptor-mediated transcytosis by using a diverse range of nano-carriers.

The BBB functions as a selective barrier to import nutrients so as to maintain neuronal survival and limit the passage of neurotoxins or infectious particles. The main element of BBB functions is the presence of dozens of tight junction proteins resulting in low para-cellular permeability (e.g. cluadins 3, 5, 12, ZO1 and occludin). However, several subsidiary mechanisms also maintain the appropriate BBB functions in physiological conditions including (1) high Transendothelial Endothelial Electrical Resistance (TEER) (2) low transcytosis/pinocytosis rates (3) lack of fenestrations in the apical surface of brain microvessels (4) size selectivity for diffusion of small molecules (e.g. lipophilic small molecules, O2, and CO2). (5) carrier-mediated transport of larger nutrients such as glucose, amino-acids, ketones, nucleosides and neurotransmitters (6) receptor-mediated transcytosis of specific proteins (e.g. transferrin, or insulin) (7) and efflux of toxic metabolites, xenobiotics, and chemo-agents (56). In the TME of GBM however, the BBB disruption is due to the following cellular or molecular mechanisms: (1) imbalance of tight junction proteins due to alterations in the synthesis, trafficking, or post-transcriptional modifications (2) secretion of multiple pro-inflammatory cytokines (3) extensive edema and increased interstitial fluid pressure (4) lower capability of tumor-reactive astrocytes to support normal BBB functions (5) active degeneration of the BBB tight junction proteins by invasion of glioma cells to tumor ECs (6) leaky and haphazard nature of glioma microvessels with suboptimal delivery functions (i.e. to deliver chemoagents or TKIs). Accumulating evidence suggests that the impaired BBB in GBM pathogenesis provides an important area of research to enhance drug delivery strategies for BBB penetration. This section is devoted to a detailed and critical literature review on the BBB targeting strategies previously reported. (1) Passive targeting: Nano-carriers (NCs) can passively target neoplastic tissues through Enhanced Permeability and Retention (EPR) effect. EPR and passive targeting is highly dependent NCs characteristics such as size, shape, spatial characteristics and surface charge as well as the tumor biology, itself. Previously, numerous passively-targeted NCs have been commercialized comprising Doxil™, Abraxane™, Marqibo™, DaunoXome™, and Onivyde™ in the US; Myocet™ and Mepact™; Genexol-PM™; and SMANCS™ however, there is a rising tendency to increase the accumulation rates and enhance tissue-specific-targeting by active strategies. (2) Active targeting: several Moieties can be used to actively deliver the siRNA cargo through the BBB, including receptor substrates, cell-penetrating peptides, mAbs, aptamers, monosaccharides, polysaccharides, proteins, peptides and surface modifications. Receptor-Mediated Transcytosis (RMT) is one of the most frequently used strategies to transfer the cargos of interest into the brain. The most common RMT targets are the transferrin receptor (TfR), low-density lipoprotein (LDL) receptor, insulin receptor, ApoE receptors growth factors, biotin-binding proteins, insulin, lactoferrin, and EGFR variants (**Figure 4**).

## RNA Interference: A Future Perspective for GBM Anti- Angiogenic Therapy

Small interfering RNAs (siRNAs); known as 20-25 base pairs-in-length double-stranded non-coding RNAs; interfere expression of mRNAs. They are known as post-transcriptional silencers of a specific gene target by assembly of the RNA-induced silencing complex (RISC). This causes cleavage of the target mRNA molecules and its further degradation by cellular exonucleases (57). Molecular therapy using siRNA has indicated promising results in treating diseases caused by abnormal gene overexpression or mutation-based diseases. Naked siRNAs are unstable, and their physicochemical features (e.g. size and charge) may prevent them from crossing the BBB and the blood-tumor barrier (BTB). Additionally, they can potentiate immune responses when systemically administered and also may be entrapped by the reticuloendothelial system. Meanwhile, siRNAs loaded in tumor-targeted nanoparticles display many benefits, including minimal recognition by the immune system, more blood stability, high specificity and low off-target effects. Consequently, nanotechnology could aid development of novel and effective delivery systems that can enhance targeted delivery siRNAs and also protect them from degradation, rapid cellular washout and systemic clearance. When loaded in nano-carriers as a nanopelex, they are advantageous to mAbs/TKIs in several aspects as follows: (1) Firstly, siRNAs can be encapsulated into various nano-vehicles to mediate active transport of the nano-vehicle-siRNA complexes (nanoplexes) to targeted cells. Hence, nanoplexes may show higher tumor penetrance compared to intact mAbs (2) the nanovehicles’ characteristics can be engineered to pass through the BBB more efficiently (e.g., by consumption of a BBB-penetrating peptide which mediates active transport of nano-vehicle) (3) circulation time, pharmacodynamics and pharmacokinetics of the nanoplexes is modifiable (4) Developing multidisciplinary treatment strategies (e.g., loading multiple siRNAs, chemo agents and also radio-active isotopes into a single nano-vehicle to increase the synergic potential) (5) siRNAs have relatively lower systemic adverse events due to precise tumor targeting (e.g. by loading tumor-specific Abs on the nanoparticle’s surface). (6) Sustained and long release of siRNAs from engineered nano-vehicles reduces the injection frequency and increases the treatment efficacy (7) High selectivity of targeted nanoplexes to target a specific organ as well as a specific gene target reduces “off-target effects” compared to TKIs (8) need for relatively lower doses due to slow and targeted release which reduces tumor acquired resistance. To date, various carriers have been used for siRNA delivery to combat the main obstacles in GBM comprising liposomes, polymeric nanoparticles, gold nanoparticles and exosomes. Up to the present, only two clinical trials have been published on siRNA therapy for solid tumors (e.g. glioma), which are summarized in **Table 4**. Delivery of EphA2 siRNA *via* neutral liposomes (1,2-dioleoyl-sn-glycero-3-phosphatidylcholine or DOPC) in patients with advanced/recurrent neoplasms showed an acceptable safety profile. Moreover, delivery of Bcl2L12 siRNA-conjugated with gold nanoparticles penetrating BBB were promising and showed minimal toxicity (58, 59). Herein, we highlight the recent advances developing nano-carriers for siRNA delivery to GBM.

**Table 4:** Clinical trials on siRNAs for GBM.

SiRNA complex	Study phase	summary	Ref.
DOPC-encapsulated EphA2 siRNA	I	This first phase study examines the side effects and best dose of EphA2 siRNA in the treatment of patients with metastatic solid tumors or recurrent cases. DOPC-encapsulated siRNA slows the growth of tumor cells by targeting EphA2.	(58)
Bcl2L12 siRNA conjugated with gold nanoparticles	0	A potential treatment for GBM involves the use of RNA-interfering spherical nucleic acids that penetrate the brain and consist of nuclei of gold nanoparticles covalently bonded to small interfering RNA (siRNA) oligonucleotides.	(59)

## Nanovehicles for siRNA Delivery Across the BBB

**Figure 5:**
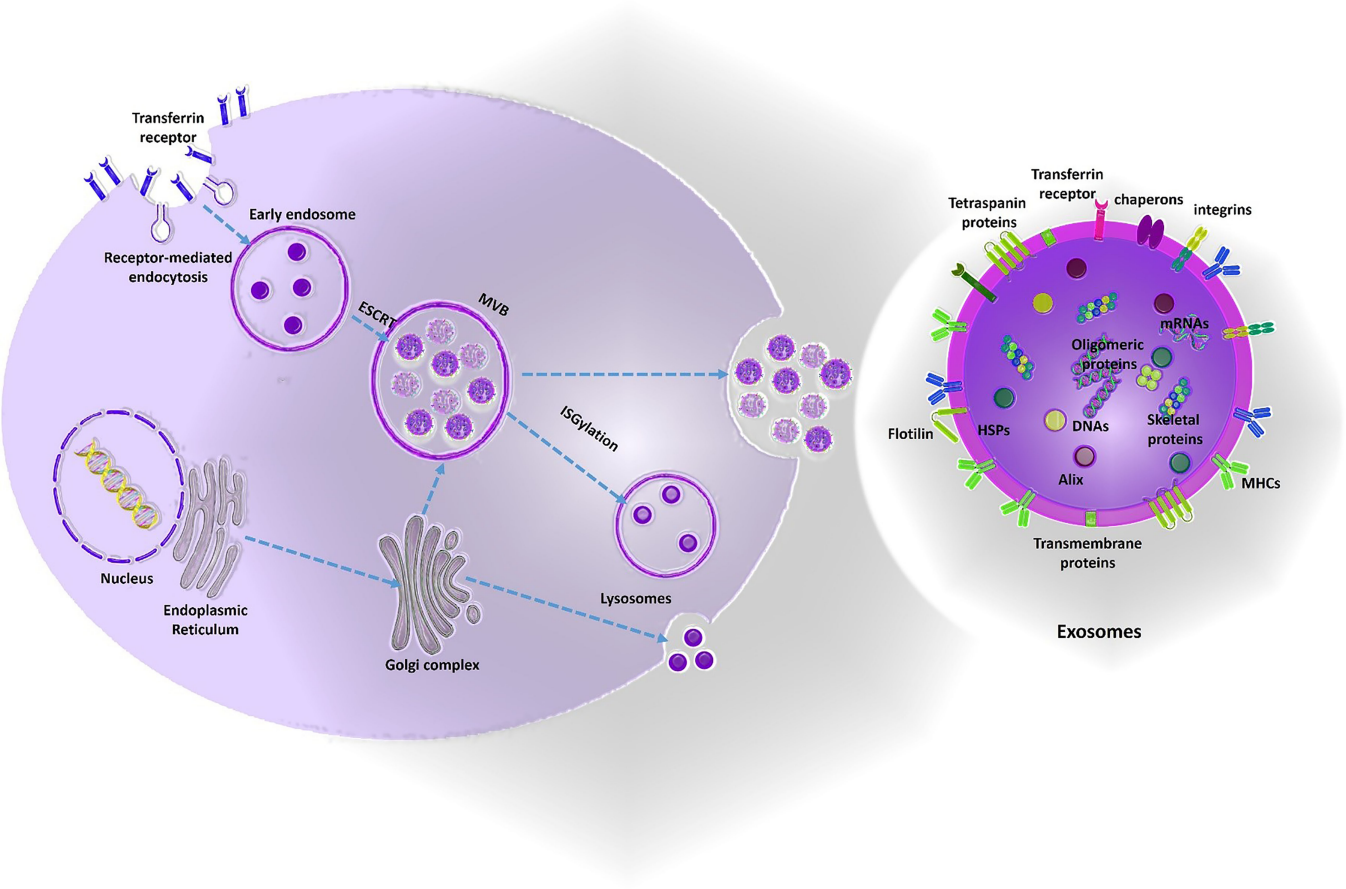
Exosomes biogenesis. Mainly, exosomes are produced by ESCRT- dependent mechanisms from early endosomes into the multivesicular bodies (MVBs). Exosomes contain the members of tetraspanin protein family and ESCRT proteins and their accessory proteins as positive markers for EV characterization. Moreover, exosomes also contain signaling cargos which mediate paracrine interaction of cells (DNAs, proteins, and also RNAs). Exomes are potent engineerable biological nano-carriers which pass through the BBB and can be used as a trojan horse to deliver the drugs to the brain. Some of the vectors used to design this figure were downloaded from Vecteezy under a free license.

The synthesis strategy, size/charge optimization or conjugation strategies should be modulated for generating BBB penetrating nano-carriers with robust drug delivery properties. Previous literature suggests nano-carriers can be designed to pass through the BBB successfully (e.g., exosomes (60), liposomes (61–63), and even gold nanoparticles (64–66). An optimal nano-carrier for delivery of siRNAs should have the following characteristics; (1) protection drugs from degradation (2) low-immunogenic properties (3) high uptake rates by target cells (4) acceptable blood circulation time (5) rapid and high accumulation at target organs (6) a long and controlled release pattern to obtain a permanent and effective gene-silencing response. Potential challenges of using synthetic nano-carriers also comprise (1) low blood circulation time (2) rapid entrapment in filter organs and recognition by the immune system causing fast clearance and (3) immunogenicity. Consequently, various strategies have been utilized to prolong nano-carriers’ circulation time in the peripheral blood (e.g. by grafting biocompatible hydrophilic polymers like polyethylene glycol or PEG). PEGylation, impairs the opsonin proteins binding to nano-carriers and protects them from recognition by reticuloendothelial system. Moreover, PEGylation can effectively prevent nanoparticles from aggregation and the endosomal release due to its esteric barrier nature and also lowers the immunogenicity of the synthetic nanocarriers. Hence, the nano-carriers can accumulate in the tumor milieu in higher concentrations. Herein, we discuss the previous attempts using nanocarriers for siRNA delivery to GBM. Previously reported nanocarriers for siRNA delivery to GBM comprise the following:

(1) Cationic liposomes: One of the most frequently used nano-carriers for gene delivery is liposome. The liposome-siRNA complex is also named as a lipoplex. Due to the negatively-charged nature of oligonucleotides, cationic lipids have attracted much attention and have advantageous properties for optimization of gene delivery process in cancers which comprise (1) easy synthesis (2) surface-modifiable domains which facilitates engineering and targeting properties (3) high and efficient loading of nucleic acids through electrostatic interactions (4) the excess cationic coats also facilitate vectors binding to negatively charged cell membranes (5) interruption of endosomal membrane to improve cytoplasmic delivery of nucleic acids. Major hurdles that limits the advantages of lipoplexes for high yield siRNA delivery comprise (1) aqueous instability of suspensions limiting the shelf life of produced siRNA-nanoparticles (2) electrostatic attraction force also is a challenge for optimized synthesis and design of lipoplexes which directly impacts the therapeutic efficacy. A balance of strong enough to protect nucleic acids from degradation during transportation and weak enough to allow for timely release of the payload of nucleic acids within target cells should be maintained. Mounting the previous evidence, cationic liposomes functionalized with two receptor-specific peptides, including Angiopep-2 and neuropilin-1 has been developed for glioma targeting and BBB penetration, respectively. They reported successful knockdown of VEGF and inhibition of glioma growth by loading VEGF-siRNA and docetaxel in the Angiopep-2 and neuropilin-1 targeting liposomes (67). Wei et al. developed an effective siRNA delivery system through T7 peptide-conjugated cationic liposomes (named as T7-LPC/siRNA NPs) as a targeted drug delivery system for transferrin receptor-mediated active targeting for GMB therapy (68). Another hurdle in using liposomes for gene delivery purposes is the loading efficacy. Development of hybrid nano-systems or introducing different alkyl chains in the same lipid with varying lengths in the hydrophobic domain are possible strategies to overcome the loading efficacy and transfection challenge. (2) Polymeric nanoparticles: Polymeric nanoparticles offer high yield transfection and are advantageous for many reasons comprising unlimited gene packing and the ability of polyplexes to be extensively modified *via* multiple modifiable moieties. In addition, polymers with specific functional groups such as positively charged or pH-sensitive moieties promote the endosomal escape of encapsulated therapeutic agents into the cytoplasm. Among the cationic polymers previously suggested for targeted drug delivery to brain, Poly (lactic-co-glycolic acid) PLGA, poly (glycolic acid) PGA, and poly (lactic acid) PLA are frequently reported. PLGA and PLA has been approved by the US Food and Drug Administration (FDA) for clinical use in humans therefore can be considered as a safe option for brain drug delivery. Kozielski et al. developed a biodegradable polymer consisting two monomers (1) bis(2-hydroxyethyl) disulfide (BR6) and (2) 4-amino-1-butanol (S4) to deliver siRNAs targeting GBM-promoting genes (e.g. Survivin, EGFR, NKCC1, YAP1, and Robo1) with promising results (69). Polyamidoamine (PAMAM) dendrimers are one of the most frequently used cationic polymers, which comprise of a variety of surface functional groups (-OH, -COOH, -NH2). Moreover, these nanoparticles has a pH-sensitive property due to the existence of protonated amine groups in an acidic condition, which imparts electrostatic repulsion between the polymer chains. These attractive features provide a versatile carrier for controlled siRNA delivery in GBM. One example was a PAMAM-dendrimer carrier with a RGD receptor-specific anchored to the surface, which was designed for the delivery of siRNA plus doxorubicin (DOX) against GBM. Peptides containing an arginine-glycine-aspartic acid (RGD) can be detected by the integrin receptors, especially ανβ3, which are often overexpressed in tumor cells, but rarely identified in normal tissue cells. Previous evidence suggested that these pH-sensitive effectively penetrate the BBB and co-deliver Doxorubicin and c-Myc-siRNAs in order to suppress GBM progression (70). Gold nanoparticles: Gold nanoparticles are reliable drug delivery systems for loading siRNAs conjugated covalently or by electrostatic conjugation onto their surface. The free thiol groups also can be used for surface modifications and bio-conjugation of targeting moieties to gold nanoparticles. Promising results have been reported by using synthetized gold-liposome nanoparticles functionalized with ApoE and rabies virus glycoprotein (RVG) as the targeting peptides for brain delivery (71). Solid Lipid Nanoparticles (SLNs): SLNs are also considered as promising nano-carriers for gene delivery because of their lipid nature, biodegradability and bio-compatibility. Due to their promising properties for oligonucleotide delivery, SLNs have been used for generation of the COVID19 vaccine; Pfizer recently. Neves et al. prepared a solid lipid nanoparticles (SLNs) functionalized with ApoE to improve brain drug delivery. Confocal images and flow-cytometry results indicated an increase in brain cellular uptake compared to the non-conjugated SLNs (72). Super-paramagnetic iron oxide nanoparticles (SPIONPs): SPIONPs have a wide range of clinical utilities (e.g. theranostic applications and hyperthermia). When external magnetic field is applied, SPIONPs can be used for efficient and targeted delivery of the loaded cargos with potential advantages such as high stability and increased blood circulation. Previously, EGFR-conjugated superparamagnetic iron oxide nanoparticles (SPIONPs) were loaded with survivin siRNAs (apoptosis-related inhibitors) and doxorubicin with promising results (73). Biological nanocarriers such as exosomes: Exosomes are natural drug delivery systems (40–100 nm) with advantageous properties for either active or passive targeting secreted by various cell types and are able to transfer different types of biological molecules (e.g. mRNAs and small RNAs). They can be obtained from autologous dendritic cells (DCs), Chimeric Antigen Receptor T cells (CAR T cells), stromal/stem cells (bone-marrow derived mesenchymal stem cells, placenta derived mesenchymal stem cells or adipose tissue mesenchymal stem cells), Natural Killer Cells (NK cells), CAR NK cells or NK T cells. To focus on the biogenesis process, one of the major exosome formation mechanisms is the endosomal sorting complexes required for transport (ESCRT) pathway however ESCRT-independent pathways may also be responsible for EV formation. Consequently, ESCRT proteins and their accessory proteins (Alix, TSG101, HSC70, and HSP90β) are noted as positive EV markers for characterization however the Tetraspanin transmembrane proteins family (e.g. CD63, CD81, and CD9) are also of the frequent markers used for EV characterization and validation of EV purification procedures (**Figure 5**) (74). Encapsulated drugs in exosomes have demonstrated multiple advantages compared to synthetic nanoparticles, including (1) more biocompatibility due to human-derived nature and better membrane fusion (2) unique proteo-lipid structure which stabilizes exosomes in blood circulation and increases their shelf-life (3) minimal recognition by the mononuclear phagocytic system (MPS) and immunogenicity (4) easy and fast production from cell culture media; a byproduct of cell-therapy facilities (5) intrinsic anti-cancer cargos of exosomes derived from mesenchymal cells or dendritic cells (e.g., miRNAs, proteins, mRNAs, or DNA fragments). Added to the mentioned advantages, exosomes may have the ability to cross different biological barriers (e.g. the BBB) (75). Despite the potential advantages of exosomes as biological nanocarriers, scale up process for high yield and purified exosome production faces many potential challenges. Major industrial-Scale exosome isolation methods depend on ultracentrifugation and ultrafiltration and both methods are time-consuming and have low yield for clinical applications (76). Mounting the previous evidence, Erviti et al. prepared engineered Lamp2b expressing self-derived dendritic cell exosomes for siRNA delivery to target the neuron-specific RVG peptide which is preferably expressed in the neurons, microglia, oligodendrocytes in the brain leading to a knockdown rate of 60% at mRNA level and 62% at protein-level for BACE1 (77). Another hurdle to use exosomes for siRNA delivery is the loading efficacy which does not exceed 20-30% in most of the previous studies. Modifying the siRNAs by adding hydrophobic tags (e.g. cholesterol tags) is a potential strategy to overcome the loading efficacy challenge (78).

## Conclusions

### Next-Generation Anti-Angiogenic Therapies

Tumor angiogenesis is a vital mechanism for maintaining tumor cell survival, providing nutrients, and oxygen uptake. Inhibition of tumor angiogenesis can act as a highly effective mechanism to combat vascular-rich tumors such as GBM. So far, extensive research has been focused on the production of novel anti-angiogenic drugs for gliomas, most of them were focused on mAbs and TKIs. Clinical application of intact mAbs faces many challenges, including low penetration into solid tumor tissues, failure to cross the BBB due to its large size, and systemic side effects. Despite the advantages of TKIs compared to mAbs (i.e., including smaller size and targeting angiogenesis *via* several molecular pathways), they also have disadvantages such as low selectivity. TKs are expressed in numerous cell types and systemic inhibition of TKs increases the risk of major systemic toxicities. Moreover, acquired resistance to anti-angiogenic TKIs is another challenge which limits their clinical advantages as adjuvants to radiation therapy or chemotherapy as a post-surgical management strategy (79). As a future perspective, siRNAs are potent effective silencers of tumor angiogenic gene expression for GBM. Exosomes obtained from various cell sources comprising autologous dendritic cells (DCs), Chimeric Antigen Receptor T cells (CAR T cells), stromal/stem cells (bone-marrow derived mesenchymal stem cells, placenta derived mesenchymal stem cells or adipose tissue mesenchymal stem cells), Natural Killer Cells (NK cells), CAR NK cells or NK T cells can serve as potent biological nano-carriers for efficient and targeted drug delivery to GBM with modifiable surface characteristics for a precisely targeted therapy with minimal systemic adverse side effects (80). Moreover, exosomes can also be used to minimize the AEs of chemo agents by targeting them directly to the tumor site with priorities to synthetic nanocarriers such as liposomes due to their human-derived and biological nature (81). Additionally, using nanobodies as an immunotaregting strategy could aid deliver the cargo of interest to tumor site with minimal systemic adverse events and optimal passage through the BBB (82).

### Combination Therapy; a Future Perspective to Improve Anti-Angiogenic Therapy Efficacy

As another future perspective, adjuvant combination immunotherapies may aid increase the efficacy of anti-angiogenic therapy for GBM which comprise immune check-point blockade (83) and DC vaccination. Moreover, using anti-angiogenic agents which target multiple downstream angiogenic signaling pathways or using a combination of antiangiogenic agents may help reduce therapy resistance and also using long-release nanoparticles to maintain the antiangiogenic properties at a low permanent concentration rather than using high dose interrupted systemic injections of mAbs/TKIs may also be effective strategies to overcome therapy resistance (84, 85). Combination therapies of antiangiogenic agents with chemoagents are also a future perspective to obtain a permanent anti-tumor response (86). In addition, anti-angiogenic therapy could serve as a potential complementary treatment adjuvant to many anti-cancer immunotherapies comprising DC therapy or adoptive T/CART cells transfer. Previous literature suggests that there is a substantial relationship between tumor aberrant angiogenesis and cytotoxic T cells functions as well as dynamics of DC maturation. Anti-angiogensis therapy empowers anti-tumor immune response and therfore using anti-angiogenic agents as adjuvants to immune therapy may be a future perspective for more efficient treatment of the GBM. In addition to increasing knowledge about developing safe and efficient nano-carriers for effective siRNA delivery, the identification of novel strategies to overcome the BBB hurdle such as using focused ultrasound will also enhance our ability to target GBM (87). Generating multidisciplinary nanoplexes delivering anti-cancer agents (e.g. chemoagents) and antiangiogenic therapeutic cargos could be a future perspective to combat glioma angiogenesis with minimal systemic adverse events and high potency in the future. Up to present numerous multidisciplinary nanoplexes are being tested in preclinical grades which could serve as potential clinical-grade next-generation anti-angiogenic therapeutics for GBM (88–98). Future work will shed light to the clinical applicability of active targeting using RMT to overcome the BBB hurdle for GBM drug delivery (99–103).

### Optimizing Carrier Design for Potent Drug Delivery Through the BBB

Despite accumulating preclinical evidence on designing BBB penetrating vehicles, still a major hurdle for successful delivery of anti-angiogenic agents to brain in clinical settings is the BBB. Despite accumulating preclinical evidence on designing BBB penetrating vehicles, still a major hurdle for successful delivery of anti-angiogenic agents to brain in clinical settings is the BBB. Up to present, numerous strategies have been introduced to overcome the BBB hurdle comprising: Convection enhanced delivery (CED), chemical permeation using vasoactive agents and hyperosmotic Manitol, physical permeation by applying external magnetic fields for magnetic vehicles, using intraoperative drug-coated wafers or intraventricular injections. However, a major shortcoming for the aforementioned methods is the infection risk. One of the most promising methods which could serve as a future prospect is active targeting *via* RMT. Future work will shed light to the clinical applicability of active targeting using RMT to overcome the BBB hurdle for GBM drug delivery.

## Author Contributions

PS and SL conceptualized and wrote the manuscript. FHA and NE also participated in writing up the paper. AZ guided the surgical aspects. MA and DA conceptualized, supervised and wrote the manuscript. All the authors have read and approved the final draft.

## Conflict of Interest

The authors declare that the research was conducted in the absence of any commercial or financial relationships that could be construed as a potential conflict of interest.

## Publisher’s Note

All claims expressed in this article are solely those of the authors and do not necessarily represent those of their affiliated organizations, or those of the publisher, the editors and the reviewers. Any product that may be evaluated in this article, or claim that may be made by its manufacturer, is not guaranteed or endorsed by the publisher.
